# Concomitant Glenohumeral Pathologies in Patients with Acromioclavicular Joint Dislocations: How Do Acute and Chronic Instabilities Differ?

**DOI:** 10.3390/jcm13061723

**Published:** 2024-03-17

**Authors:** Philipp Vetter, Manije Massih, Frederik Bellmann, Larissa Eckl, Philipp Moroder, Asimina Lazaridou, Markus Scheibel

**Affiliations:** 1 Department of Traumatology, University Hospital Zurich, Rämistrasse 100, 8091 Zurich, Switzerland; 2 Department of Locomotive Surgery, Vivantes Clinic, Neue Bergstrasse 6, 13585 Berlin, Germany; 3 Department of Shoulder and Elbow Surgery, Schulthess Clinic, Lengghalde 2, 8008 Zurich, Switzerland; 4 Center for Musculoskeletal Surgery, Charite-Universitaetsmedizin, Augustenburger Platz 1, 13353 Berlin, Germany

**Keywords:** acromioclavicular joint instability, concomitant glenohumeral pathologies, arthroscopically assisted surgery

## Abstract

**Background:** Concomitant glenohumeral pathologies may be present in patients with acromioclavicular joint (ACJ) dislocations. This study aims to record and compare the prevalence and treatment of CGP in cases with acute and chronic ACJ dislocations. **Methods:** This retrospective cross-sectional binational, bicentric study included patients that underwent arthroscopically assisted stabilization for acute (group A) and chronic (group C) ACJ dislocations. Intraoperatively, CGPs and eventual treatments (debridement and reconstructive measures) were recorded. **Results:** The study included 540 patients (87% men; mean age 39.4 years), with 410 (75.9%) patients in group A and 130 (24.1%) in group C. Patients in group C were older (*p* < 0.001). The CGP prevalence was 30.7%, without a difference between groups A and C (*p* = 0.19). Supraspinatus tendon (SSP) and labral lesions were most common. Within group C, CGPs were more prevalent in surgery-naïve patients (*p* = 0.002). Among 49 patients with previous surgical treatment, CGPs tended to be more common in patients with prior open surgery than arthroscopically assisted surgery (*p* = 0.392). Increased CGP prevalence was associated with higher age (r = 0.97; *p* = 0.004) (up to 63% in the oldest age group, but also 17% for youngest age group) and higher in cases with Rockwood type-IIIB injuries compared to type-V injuries (*p* = 0.028), but type-IIIB injuries included more group C cases (*p* < 0.001). The most frequently found CGPs were treated by debridement rather than reconstructive interventions (SSP and labrum: *p* < 0.001, respectively). **Conclusions:** This study shows that one in three patients with ACJ instabilities has a CGP, especially elderly patients. Most of the CGPs were treated by debridement rather than constructive interventions.

## 1. Introduction

Acromioclavicular joint (ACJ) dislocations are one of the most common injuries of the shoulder girdle [1], usually classified according to the radiologic six-grade classification by Rockwood (RW) [2]. Sprains and minor dislocations (RW types I–II) are usually treated non-operatively, whereas high-grade instabilities (RW types IV–VI) conventionally require surgical intervention [3]. Concerning RW type-III injuries, recent evidence favors non-operative treatment [4,5,6,7,8].

The time-to-surgery interval after injury can be classified into an acute (0–21 days) or chronic (>21 days) phase [9,10,11,12,13,14].

For surgical treatment in the acute setting, synthetic stabilization is regularly performed [15,16] and may be augmented by an acromioclavicular (AC) cerclage [14,17] or tendon graft [17,18,19,20]. In the chronic setting, the use of tendon grafts is recommended [20,21,22]. Independent of the interval since injury, coracoclavicular and AC ligament addressment is essential to restore bidirectional ACJ stability [23,24].

Coincidental observations of concomitant glenohumeral pathologies (CGP) in ACJ dislocations led many authors to advocate for glenohumeral arthroscopy prior to ACJ stabilization to address the CGP [9,10,11,12,13,25,26,27,28,29]. This may be particularly reasonable in light of residual symptoms after ACJ treatment, especially after non-operative treatment [8,30,31,32,33,34].

After previous studies on CGPs in acute ACJ dislocations, a recent study suggested that chronic cases were associated with a higher prevalence of CGP, occurring in up to 53% of cases [26]. Regardless of this finding, the (type of) treatment for the CGP (debridement vs. reconstruction) has been poorly described before.

The aim of our study was to describe and compare the prevalence of CGPs in patients with acute and chronic ACJ dislocations (RW types II–V) undergoing arthroscopically assisted ACJ stabilization and to investigate the treatment for CGPs.

## 2. Materials and Methods

Databases of two institutions (Schulthess Clinic, Zurich, Switzerland; Charité Universitaetsmedizin, Berlin, Germany) were utilized in our analysis. Ethical approval was granted (BASEC-Nr. 2020-00464).

In this retrospective, cross-sectional, binational and bicentric study, all patients who underwent arthroscopically assisted stabilization for acute and chronic ACJ instabilities (RW types II–V) between February 2007 and April 2019 were included consecutively.

We excluded patients with incomplete data and prior fractures, arthroplasty or tendon transfers of the shoulder girdle. 

ACJ dislocations were classified radiographically [2,4]. Differentiation between RW types IIIA/IIIB was also performed radiographically according to the presence (IIIB) or absence (IIIA) of an overriding of the clavicle on the acromion [4]. 

Patient parameters (age at time of surgery, gender, injury-to-surgery-interval, RW type, and previous surgery with respective type being open/arthroscopically assisted) were extracted from the particular clinic patient information system and coded into a centralized project database.

According to the injury-to-surgery-interval, patients were divided into two groups: acute (group A; 0–21 days) and chronic ACJ dislocations (group C; >21 days) [9,10,11,12,13,14]. Within group C, patients were divided into one group with previous surgical treatment for the ACJ dislocations and one without prior surgical treatment. Cases with previous surgical treatment were further differentiated by the respective type of prior interventions being open or arthroscopically assisted. According to patient age, several groups were formed (18–30; 31–40; 41–50; 51–60 and 61–71 years).

During surgery, a diagnostic glenohumeral arthroscopy was performed via a standard posterior viewing portal prior to the ACJ reconstruction in all cases. Relevant glenohumeral structures for evaluation included the rotator cuff (RC) [8,9,35,36] (Fox and Romeo classification [35]: type I: partial thickness tear; type II: complete tear of the upper 25 % tendon portion; type III: complete tear of the upper 50 % tendon portion; type IV: complete rupture of the tendon portion. Snyder classification of SSP lesions [36]: A: articular-sided tear; B: bursal-sided tear; C: complete tear), the labrum (Kim’s lesion: incomplete or concealed posterosuperior labrum lesion [37]; glenolabral articular disruption, GLAD [38]), the long head of the bicep tendon (LHB) and its anchor (superior labrum anterior and posterior, SLAP, according to Snyder [39]: type I: frayed and degenerated superior labrum; type II: superior labrum and bicep tendon detached from glenoid rim; type III: SLAP lesion with a bucket-handle tear of the superior labrum, while the remaining labrum remains attached to the glenoid rim; type IV: SLAP lesion with an extension of the bucket-handle labrum tear into the biceps tendon), the pulley system (Habermeyer classification [40]: type I: isolated lesion of the superior glenohumeral ligament, SGHL; type II: lesion of the SGHL and a partial, articular-sided SSP lesion; type III: lesion of the SGHL and a partial, articular-sided subscapularis tendon lesion; type IV: lesion of the SGHL with a partial articular-sided lesion of the SSP and subscapularis) and the glenohumeral joint surfaces. Subacromial arthroscopy was only performed if anamnesis (also regarding symptoms prior to accident, e.g., impingement) or clinical findings (e.g., impingement/rotator cuff test) were suggestive for such.

These anatomic structures were routinely screened for possible CGPs and, if deemed relevant by the attending surgeon (for example, and trending towards high-grade lesions of the rotator cuff or labrum), treated during the same surgery. All findings and interventions were documented in the surgery reports. Reports were screened for CGPs and respective treatments, which were generally classified as debridement or reconstruction. If available, arthroscopic images were additionally evaluated by two independent examiners (M.M., M.S.) to double-check for pathologies. The character of the CGP was classified as either acute/traumatic or chronic/degenerative [12], to the extent possible. 

### Standard Analysis

Statistical analysis was executed using R software (v4.0.3). All study parameters were described using standard statistics including range and ratios, where appropriate. Age is reported with standard deviation. Following the testing of normal distribution (Kolmogorov–Smirnov test), group comparisons were performed by use of the Mann–Whitney-U test. The Chi-Square test (or Fisher’s exact test, depending on sample size) was utilized to compare prevalences between groups, and, if appropriate, to compare the odds ratio (OR). For correlation analysis, the Pearson coefficient or the Spearman coefficient were calculated according to scale level (continuous vs. ordinal). Results were considered significant if *p* < 0.05.

## 3. Results

### 3.1. Patient Cohort

**Figure 1:**
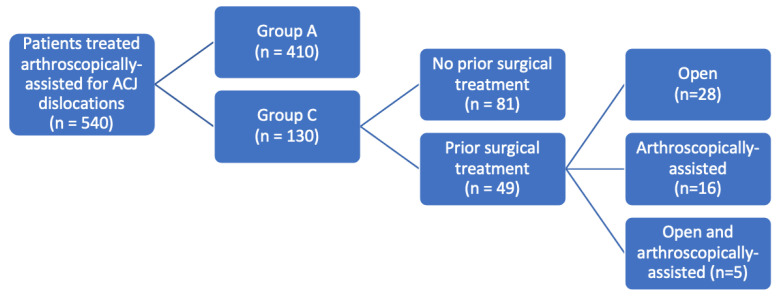
Flowchart of the study cohort according to injury-to-surgery-interval and, for group C, previous treatment type. A, acute. ACJ, acromioclavicular joint. C, chronic.

A total of 540 patients (87% men; mean age 39.4 ± 12.1 years) were included in the study, comprising 410 (75.9%) patients in group A and 130 (24.1%) in group C (Figure 1). Patients in group A underwent surgery after a mean injury-to-surgery-interval of 9 ± 4 days (range, 1–21 days) while the patients in group C underwent surgery after a mean injury-to-surgery-interval of 277 ± 201 days (range, 22–874 days). Patients in group C were older than patients in group A (43.8 ± 12.6 years compared to 38.0 ± 11.7 years; *p* < 0.001). 

Within group C, 37.7% (n = 49) had received prior surgical treatment. Of these 49 patients, in turn, 28 were previously treated openly (57.1%) and 16 patients (32.7%) were treated in a way that was arthroscopically assisted. Five patients had previous surgeries using both methods.

ACJ dislocations were classified as type II in 12 patients (2.2%), type III in 165 patients (30.6%) [2 cases with IIIA (0.4%) and 163 with IIIB (30.2%)], type IV in 7 patients (1.3%) and type V in 356 patients (65.9%).

### 3.2. Incidence and Type of Concomitant Glenohumeral Pathologies

We identified CGPs in 30.7% of all cases (n = 166), with no significant difference observed between group A (29.3%, n = 120) and group C (35.4%, n = 46) (*p* = 0.192). The majority of CGPs were characterized as low grade (Snyder Type A1 [36], Fox and Romeo type I [35]) (Table 1). Among all CGP cases, 7.2% (n = 12) were defined as acute/traumatic, and 31.3% (n = 52) were categorized as chronic/degenerative. The classification of most pathologies remained undetermined (61.5%, n = 102). Additionally, almost half of the patients diagnosed with a CGP exhibited more than one pathology (46.4%, n = 77). The most prevalent CGPs were lesions to the supraspinatus tendon (SSP) (14.8%; n = 80) and labral lesions (14.4%; n = 78), which to the most part were SLAP lesions (11.7%; n = 63). SSP tendon lesions appeared more often in group C (21.5%; n = 28) than in group A (12.7%; n = 52) (*p* = 0.02; OR 1.89). 

CGPs were more common in patients without prior surgery [45.7% (n = 37) vs. 18.4% (n = 9); *p* = 0.002; OR 3.74], including a higher rate of SSP lesions [30.9% (n = 25) vs. 6.1% (n = 3); *p* < 0.001; OR 6.85] and LHB lesions [9.9% (n = 8) vs. 0%; *p* = 0.024].

In instances where previous surgical treatment was carried out openly (n = 28), the occurrence of CGPs was more frequent compared to cases with arthroscopically assisted treatment (n = 16) [17.9% (n = 5) vs. 6.3% (n = 1); OR 3.26]. Nevertheless, this difference did not achieve statistical significance. (*p* = 0.392).

### 3.3. Association between Concomitant Glenohumeral Pathologies and Age/Rockwood Type

A higher age was associated with a higher prevalence of CGPs (r = 0.97; *p* = 0.004) (Table 2). There was no difference in CGP prevalence by gender [men: 31.0% (146/471) vs. women: 29.0% (20/69); *p* = 0.735; OR 1.11].

Regarding the two main groups according to RW type (IIIB; V), CGPs were more common in patients with a type-IIIB injury (37.4%; n = 61) as opposed to a type-V injury (27.8%; n = 99; *p* = 0.028; OR 1.51) at a comparable age (IIIB: 38.7 ± 12.8 years vs. V: 39.6 ± 11.9 years; *p* = 0.320). However, the RW type-IIIB group included a higher rate of group C cases [41.1%; (n = 67) vs. 13.8%; (n = 49); *p* < 0.001]. SSP lesions were more common in this group [IIIB: 19.0% (n = 31) vs. V: 12.4% (n = 44); *p* = 0.045; OR 1.64). 

When separately assessing RW types IIIB and V within each group (A, C), there was no significant difference in CGP prevalence across groups [group A: IIIB: 35.4% (34/96) vs. V: 27.7% (85/307); *p* = 0.147; OR 1.43] [group C: IIIB: 40.2% (27/67) vs. V: 28.6% (14/49); *p* = 0.268; OR 1.69].

### 3.4. Treatment of Concomitant Glenohumeral Pathologies

More than two thirds (69.2%; n = 115) of all cases with CGP were deemed relevant for treatment (Table 3). Treatment was performed more frequently in group A than in group C (*p* = 0.003; OR 2.88). 

In group A, SSPs were treated more frequently (*p* < 0.001; OR 18), without any difference in the rate of reconstructive measures or debridement (*p* = 0.392).

Additional reconstructive measures were performed in 31.3% (n = 52) of all CGPs (LHB tenodesis: n = 27; RC repair: n = 20; labrum repair: n = 4; microfracturing: n = 1).

Elderly patients (61–71 years) received surgical treatment for CGPs more often than younger patients (18–30 years) [37.0% (10/27) vs. 10.2% (16/157); *p* < 0.001; OR 5.18).

The need for surgical treatment was not more frequent between men and women [men: 22.1% (104/471) vs. women 15.9% (11/69); *p* = 0.245; OR 1.49] or RW types IIIB and V (IIIB: 20.2% (33/163) vs. V: 22.2% (79/356); *p* = 0.617; OR 0.89).

Lesions of the RC (SSP/SSC) and labrum (mainly SLAP lesions) were the most common type of CGP and were treated in 60 to 78% of cases when detected (Table 4). SLAP lesions were treated in 82.5% of cases (52/63).

For SSP and labrum lesions, debridement was carried out more often than reconstructive interventions (SSP: *p* < 0.001; labrum: *p* < 0.001). There was a similar tendency for SSC lesions (*p* = 0.166).

## 4. Discussion

The aim of our study was to describe and contrast the prevalence and therapy of CGPs in patients with acute and chronic ACJ instabilities. 

Our study, featuring the largest cohort to our knowledge, demonstrated that approximately one in three patients with acute or chronic ACJ instabilities exhibits at least one CGP. Almost half of the patients presenting with a CGP were diagnosed with more than one pathology. Although most CGPs were classified as chronic/degenerative rather than acute/traumatic, most pathologies remained undetermined in their relation to trauma. Most of the CGPs were treated by debridement rather than reconstructive interventions.

CGP prevalence was lower in patients with prior surgery. There was a trend toward lower rates following arthroscopically assisted treatment, although it did not reach statistical significance.

The prevalences of CGP were higher in elderly patients. Patients with RW type-IIIB injuries had a higher rate of CGPs compared to type V but were confounded by a higher percentage of group C cases and a long injury-to-surgery-interval in the RW type-IIIB group.

Most CGPs, particularly in older patients, were identified intraoperatively by the surgeon, and 31% of all CGPs necessitated additional reconstructive surgical treatment.

Lesions of the SSP/SSC and labrum were most common and mainly treated by debridement rather than reconstructive measures. 

CGPs in arthroscopically assisted treatment of ACJ dislocations were previously described to occur in up to 53% of cases [9,10,11,12,13,25,26,27,28,29]. They were initially examined in acute cases [12,13,29], although the relation to trauma was rather unclear [12].

Arrigoni et al. [25] were the first group to compare the prevalence of CGPs in acute and chronic cases, demonstrating no difference between groups. Jensen et al. [26] extended the analysis for acute and chronic cases (cut-off for time-to-surgery interval: 21 days) and results revealed a higher prevalence of CGPs for chronic cases. Both studies documented a higher incidence of CGPs in older patients, utilizing a cut-off age of 45 years established by Arrigoni et al. [25].

The current study corroborates the high rate of CGPs (albeit mainly low-grade) in surgically treated ACJ dislocations, although no difference between groups A and C was found. Age was confirmed as a significant factor linked to the presence of (possibly asymptomatic) CGPs [41]. These CGPs can be eligible for surgical treatment in light of pain or functional reasons [42], including the consideration of eventual tear progression that can negatively affect these two aspects [43]. However, the CGPs might also be treated non-operatively initially and re-evaluated later if they would present as symptomatic in the further course (in elderly patients).

Arrigoni et al. [25] also found no difference between acute [23.4% (15/64)] and chronic cases [41.1% (14/34)], but this analysis was presumably underpowered.

We demonstrated no higher CGP prevalence in group C, in contrast to findings reported by Jensen et al. [26]. However, Jensen et al. excluded patients with previous surgery while our group C included cases with previous shoulder surgery except for fractures, arthroplasty or tendon transfers. 

Patients in group C with prior surgical treatment (37.7%) displayed a lower rate of CGPs than surgically naïve patients. Although it is presumed that CGPs were addressed during index surgery, validation would specifically apply to debridement in cases with previous arthroscopically assisted treatment. The therapeutic value such as rest, analgesia, physiotherapy or biomechanically improving surgery on the healing/subsequent absence of CGPs cannot be quantified.

Regarding RW types, Tischer et al. [29] and Jensen et al. [26] found tendencies for CGP to occur more often in types V. In our study, we found that type IIIB was linked to a higher CGP prevalence than type V cases at a similar age. However, this group exhibited a higher rate of group C cases and a longer injury-to-surgery-interval and a higher rate of SSP lesions. Missing differences in the sub-analysis of CGP prevalence within group A and C may be subject to a type-II-error. 

These observations may be interpreted as an indicative of selection bias, considering that patients in the RW type-IIIB group primarily underwent non-operative treatment in the first place. These patients may have sought re-consultation later on in the chronic setting due to persistent complaints. 

Although various aspects of residual symptoms, especially after non-operative treatments of ACJ dislocations, have been previously described [8,30,31,32,33,34], the etiologic cause for (such) re-presentation after ACJ dislocations in our study is unknown and the cross-sectional design precludes an explanation. Our study implied a potential association of residual symptoms and CGPs, as 31.3% of CGPs were subject to further intraoperative reconstructive treatment. Reconstructive measures were more frequently undertaken in in patients with chronic than acute ACJ injuries. This percentage is comparable to Jensen et al. [26].

It is unclear if CGPs were caused by the same incident as the ACJ dislocation at a similar mechanism [39,44,45,46], if they pre-existed and were possibly aggravated by the trauma or if they developed secondarily as a result of altered glenohumeral movement patterns [47,48,49,50]. The uncertainty is reflected in the fact that 61.5% of all CGPs could not be classified as chronic/degenerative or acute/traumatic. 

The use of arthroscopically assisted techniques allows simultaneous detection and treatment of CGPs. However, the value the of CGP treated (in the same session) is yet to be determined. Besides reconstructive procedures, especially the usefulness of less interfering treatment (debridement) remains largely unknown and should be subject to further research. The observation that the most common CGPs were addressed by debridement rather than reconstructive measures implied additional significance.

Several limitations should be considered before interpreting our results. The retrospective design introduces a potential for selection bias in this study, as CGPs might have led to symptoms and surgical intervention in a chronic setting. To reduce general selection bias, patients were extracted consecutively. The retrospective and descriptive study nature prevents making any implications about the etiology of CGPs or about treatment outcomes. In spite of the overall large sample number, a beta-error is assumed for the small group size of previously operated cases in group C. Subacromial arthroscopy was only performed in suspicious cases.

Despite these limitations, we were able to offer a comprehensive and large-scale study on the prevalence and type of treatment of CGPs in arthroscopically assisted ACJ stabilization.

## 5. Conclusions

This study, featuring the largest sample size to date to our knowledge, shows that one in three patients with acute and chronic ACJ instabilities has a CGP. CGPs were particularly prevalent in elderly patients, reaching almost two-thirds of cases.

Most of the CGPs were treated by debridement rather than constructive interventions.

## Figures and Tables

**Table 1:** Prevalence and subclassification of concomitant glenohumeral pathologies in the overall group and for group A and C. A, acute; C, chronic; CGPs, concomitant glenohumeral pathologies; SLAP, superior labrum anterior and posterior; GLAD, glenolabral articular disruption. Bold *p*-values indicate significant results (*p* < 0.05).

CGP	Subclassification	Overall n (%)	Group A n (%)	Group C n (%)	*p*-Value
n		540	410	130	
Overall prevalence		166 (30.7)	120 (29.3)	46 (35.4)	0.192
Supraspinatus tendon lesion		80 (14.8)	52 (12.7)	28 (21.5)	**0.016**
Supraspinatus tendon lesion type	Snyder classified	67 (83.8)	42 (80.8)	25 (89.3)	0.325
	Snyder type A1	55 (82.0)	35 (83.3)	20 (80.0)	0.118
	Snyder type A2	10 (14.9)	5 (11.9)	5 (20.0)	0.808
	Snyder type B2	1 (1.5)	1 (1.5)	0	
	Snyder type C2	1 (1.5)	1 (1.5)	0	
	Non-classified partial	2 (2.5)	2 (3.8)	0	
	Subtotal rupture	3 (3.8)	3 (5.8)	0	
	Total rupture	7 (8.8)	4 (7.7)	3 (10.7)	0.648
Subscapularis tendon lesion		36 (6.7)	26 (6.3)	10 (7.7)	0.551
Subscapularis tendon lesion type	Fox and Romeo classified	32 (88.9)	24 (92.3)	9 (90.0)	0.822
	Fox and Romeo type I	26 (81.3)	19 (79.2)	7 (77.8)	0.930
	Fox and Romeo type II	5 (15.6)	4 (16.7)	1 (11.1)	0.692
	Fox and Romeo type IV	1 (3.2)	1 (4.2)	0	
	Non-classified partial	4 (11.1)	2 (7.7)	2 (20.0)	0.460
Labral lesion		78 (14.4)	60 (14.6)	18 (13.8)	0.887
Labral lesion type	SLAP lesion	63 (11.7)	48 (11.7) 33/4/1/10	15 (11.5) 7/5/1/2	0.995
	Snyder type I	40 (63.5)	33 (68.8)	7 (46.7)	0.121
	Snyder type II	9 (14.3)	4 (8.3)	5 (33.3)	0.058
	Snyder type III	2 (3.2)	1 (2.1)	1 (6.7)	0.377
	Snyder type IV	12 (19.0)	10 (20.8)	2 (13.3)	0.518
	Bankart lesion	1 (1.3)	1 (1.7)	0	
	GLAD lesion	4 (5.1)	3 (5.0)	1 (5.6)	0.925
	Reverse Bankart lesion	3 (0.6)	2 (0.5)	1 (0.8)	0.707
	Kim’s lesion	1 (1.3)	1 (1.7)	0	
	Non-classified labral lesion	6 (7.7)	5 (8.3)	1 (5.6)	0.698
Long head of bicep tendon lesion		22 (4.1)	14 (3.4)	8 (6.2)	0.201
Long head of bicep tendon lesion type	Partial rupture	11 (50.0)	7 (50.0)	4 (50.0)	1
	Total rupture	2 (9.1)	1 (7.1)	1 (12.5)	0.674
	Instability	9 (40.9)	6 (42.9)	3 (37.5)	0.806
Pulley lesion		31 (5.7)	23 (5.6)	8 (6.2)	0.816
Pulley lesion type	Habermeyer type I	13 (41.9)	12 (52.2)	1 (12.5)	0.051
	Habermeyer type II	8 (25.8)	4 (17.4)	4 (50.0)	0.069
	Habermeyer type III	6 (19.4)	5 (21.7)	1 (12.5)	0.569
	Habermeyer type IV	1 (3.2)	1 (4.3)	0	
	Non-classified	3 (9.7)	1 (4.3)	2 (25.0)	0.089
Subacromial impingement		9 (1.7)	8 (2.0)	1 (0.8)	0.694
Chondral defects		14 (2.7)	9 (2.2)	5 (3.8)	0.302
Simultaneous glenohumeral dislocation		2 (0.4)	1 (0.2)	1 (0.8)	0.424

**Table 2:** Percentage of patients with concomitant glenohumeral pathologies in different age groups. CGPs, concomitant glenohumeral pathologies. Bold *p*-values indicate significant differences (*p* < 0.05).

Age [Years]	Number of Patients within Each Group	Number of Patients with CGPs	Percentage of Patients with CGPs	*p*-Value (vs. Younger Age Group)
18–30	157	26	16.6	
31–40	140	33	23.6	0.131
41–50	140	57	40.7	**0.002**
51–60	76	33	43.4	0.124
61–71	27	17	63.0	0.081

**Table 3:** Treatment of concomitant glenohumeral pathologies. A, acute; C, chronic; CGPs, concomitant glenohumeral pathologies; SSP, supraspinatus tendon. Bold *p*-values indicate significant differences (*p* < 0.05).

Category	Percentage (Ratio)		*p*-Value
CGP deemed relevant for treatment	69.2 (115/166)		
Group A vs. C	A	C	
-Deemed relevant for treatment	75.8 (91/120)	52.2 (24/26)	**0.003**
-Reconstructive	36.3 (33/91)	50.0 (12/24)	0.321
-Debridement	63.7 (58/91)	50.0 (12/24)	0.321
-SSP	85.7 (42/49)	25.0 (7/28)	**<0.001**
-Reconstructive	28.6 (12/42)	42.9 (3/7)	0.392
-Debridement	71.4 (30/42)	57.1 (4/7)	0.392

**Table 4:** Percentage of concomitant glenohumeral pathologies treated. CGPs, concomitant glenohumeral pathologies. Bold *p*-values indicate significant differences (*p* < 0.05).

Type of CGP	Number of Cases	Percentage of CGP Treated (n)	*p*-Value (Percentage Treatment vs. Non-Treatment)	Percentage of CGP Debrided (n)	Percentage of CGP Reconstructed (n)	*p*-Value (Debridement vs. Reconstruction)
Supraspinatus tendon	80	61.3 (49)	**0.004**	71.4 (35)	28.6 (14)	**<0.001**
Labrum	78	78.2 (61)	**<0.001**	65.6 (40)	34.4 (21)	**<0.001**
Subscapularis tendon	36	72.2 (26)	**<0.001**	61.5 (16)	38.8 (10)	0.166

## Data Availability

Data are available on request due to ethical restrictions. The data presented in this study are available on request from the corresponding author. The data are not publicly available.
